# Foot shape and radiographs of free-ranging Nubian giraffe in Uganda

**DOI:** 10.1371/journal.pone.0252929

**Published:** 2021-12-16

**Authors:** Liza Dadone, Steve Foxworth, Robert Aruho, Amy Schilz, Andrea Joyet, Myra Barrett, Peter Morkel, Garrett Crooks, Julian Fennessy, Matthew S. Johnston

**Affiliations:** 1 Veterinary Department, Cheyenne Mountain Zoo, Colorado Springs, Colorado, United States of America; 2 Zoo Hoofstock Trim Program, Equine Lameness Prevention Organization, Berthoud, Colorado, United States of America; 3 Department of Veterinary Medicine, Uganda Wildlife Authority, Kampala, Uganda; 4 Animal Department, Cheyenne Mountain Zoo, Colorado Springs, Colorado, United States of America; 5 Department of Environmental Health and Radiological Sciences, Colorado State University, Fort Collins, Colorado, United States of America; 6 Wildlife Veterinary Consultant, Karasburg, Namibia; 7 Department of Clinical Sciences, College of Veterinary Medicine and Biomedical Sciences, Colorado State University, Fort Collins, Colorado, United States of America; 8 Giraffe Conservation Foundation, Windhoek, Namibia; University of Tasmania, AUSTRALIA

## Abstract

Foot health in zoo giraffe has been a topic of recent research, although little is known about the foot health of free-ranging giraffe. This study describes the foot shape and radiographic pathological changes in 27 young adult Nubian giraffe (*Giraffa camelopardalis camelopardalis*) from a translocation in Uganda (August 2017). Giraffe feet were observed to have a concave sole, the hoof wall was longest by the toe tip, and the weight-bearing surface of the foot was primarily along the periphery of the foot including hoof wall, parts of the heel, and the edge of the sole. Radiographs showed that pedal osteitis and sesamoid bone cysts were relatively uncommon (3/24 giraffe with osteitis, 1/24 giraffe with sesamoid cysts), and that no giraffe in the study had P3 joint osteoarthritis, P3 rotation, or P3 fractures. Radiographs consistently demonstrated a positive palmar/plantar angle with the sole of the hoof thicker at the heel than by the toe tip, with the non weight-bearing palmar/plantar angle measuring 1.6°- 4.3°. This is the first systematic review of foot shape and radiographs in free-ranging giraffe and demonstrates a low prevalence of foot pathologies. This study suggests qualitative differences in foot shape, foot health, radiographic anatomy, and foot pathologies when comparing free-ranging and zoo giraffe. Further research is needed to identify why these differences occur and whether husbandry modifications could help improve zoo giraffe foot health and prevent associated lameness.

## Introduction

Hoof overgrowth and chronic lameness are well-described health concerns for zoo giraffe [1–4]. Causes of lameness appear to be multifactorial, and include diseases of the hoof [5–7], soft tissues [8, 9], joints [10–14], and bones [15–19].

To better diagnose and manage chronic lameness without restraint or anesthesia, some zoos have trained giraffe to voluntarily participate in hoof care [20]. In at least one giraffe herd trained for front foot radiographs, there was a high prevalence of osteoarthritis, pedal osteitis, pedal bone rotation, pedal bone fractures, and sesamoid bone cysts [21]. Case reports suggest some of these pathologies also occur at other zoos [12, 17, 19, 22]. While overgrown hooves and abnormal foot shape have been reported in free-ranging giraffe [23], normal foot shape and prevalence of foot pathologies in free-ranging giraffe have not been previously described.

While giraffe are commonly displayed in zoos, many wild giraffe populations are in marked decline. Overall, giraffe populations have decreased by up to 40% in the past 30 years and as a single species are listed as Vulnerable to Extinction by the International Union for the Conservation of Nature (IUCN) [24]. Historically, the giraffe subspecies in Uganda was identified as Rothschild’s giraffe (*Giraffa camelopardalis rothschildi*). Recent genetic analysis [25–28] shows that there are four distinct species of giraffe and categorizes Uganda’s subspecies as Nubian giraffe (*Giraffa camelopardalis camelopardalis*), which is how they are referenced in this paper. This updated taxonomic classification was not without initial controversy [24, 29] however, these concerns have since been rebutted [27, 28]. At time of publication, the taxonomy used in the article has yet to be reviewed by the IUCN SSC Giraffe & Okapi Specialist Group, however, is recognized by the Encyclopedia of Life [30], with its taxonomic basis linked to a partnership of the Integrated Taxonomic Information System [31] and Species 2000 Catalogue of Life [32].

In Uganda, more than 90% of the giraffe live in one national park: the north side of Murchison Falls National Park (MFNP) [33]. To help minimize the potential impact from oil and other infrastructure development occurring in and around this park, a founder population of young giraffe were translocated across the Nile River, then reintroduced to an historic habitat.

The objective of the current study was to describe foot shape, weight-bearing surfaces, and radiographic anatomy in the feet of free-ranging giraffe opportunistically evaluated during the translocation of giraffe to the south side of MFNP. A secondary objective was to describe pathological lesions identified radiographically in this population.

## Materials and methods

### Capture and restraint

Twenty-seven free-ranging giraffe were immobilized for translocation within MFNP in Uganda during the intermediate wet season in August 2017. Field captures were led by the Uganda Wildlife Authority, with additional staffing and support from the Uganda Wildlife Education Centre, the Giraffe Conservation Foundation, and zoological veterinarians.

All giraffe were apparently healthy young adults living in the predominantly savanna, Borassus palm woodland, and riverine woodland on the north side of MFNP [34]. Animals were estimated to be between 1–6 years old, with estimated weights between 300–800 kg. Each giraffe was immobilized with 8–9 mg of Etorphine Hydrochloride (Captivon 98®, 9.8 mg/ml, Wildlife Pharmaceuticals Pty Ltd, South Africa) combined with 40 mg Azaperone Tartrate (Azaperone, 100 mg/ml, Kyron Laboratories Pty Ltd, South Africa) in a single dart. Animals were darted from a vehicle in the shoulder or thigh muscles, using a Dan-Inject rifle (Dan Inject Mod 9618., Daninject, Denmark). Once in lateral recumbency, giraffe were restrained by the capture team, blindfolded, and immediately given 16–18 mg Diprenorphine Hydrochloride reversal (Activon®; 12 mg/ml, Wildlife Pharmaceuticals Pty Ltd, South Africa). Most giraffe also were given Doxapram (21 giraffe received 100 mg i.v., one received 200 mg i.v., five giraffe received none; Dopram® 20 mg/ml, Hikma Pharmaceuticals USA Inc., Eatontown, NJ, U.S.A.).

Giraffe were maintained in lateral recumbency until loading into a recovery trailer and subsequently into a boma before translocation. For human safety, a leg rope was placed on the giraffe before collecting data on the feet.

### Radiographs

The feet of 24 giraffe were radiographed, from five males and 19 females. Three additional animals were not imaged radiographically because of field conditions such as heavy rain. Of the 86 feet that were imaged, 45 were front feet and 41 were hind feet. Twenty giraffe had radiographs of all four feet, one male had only front feet imaged, one female had one front and one hind foot imaged, and one male and one female each had only one front foot imaged due to field conditions. To help minimize procedure time, typically only one radiograph of each foot was collected. While orthogonal views would have been ideal, the feet were radiographed using an oblique projection that shows multiple radiographic foot pathologies in some zoo giraffe [21].

**Fig 1:**
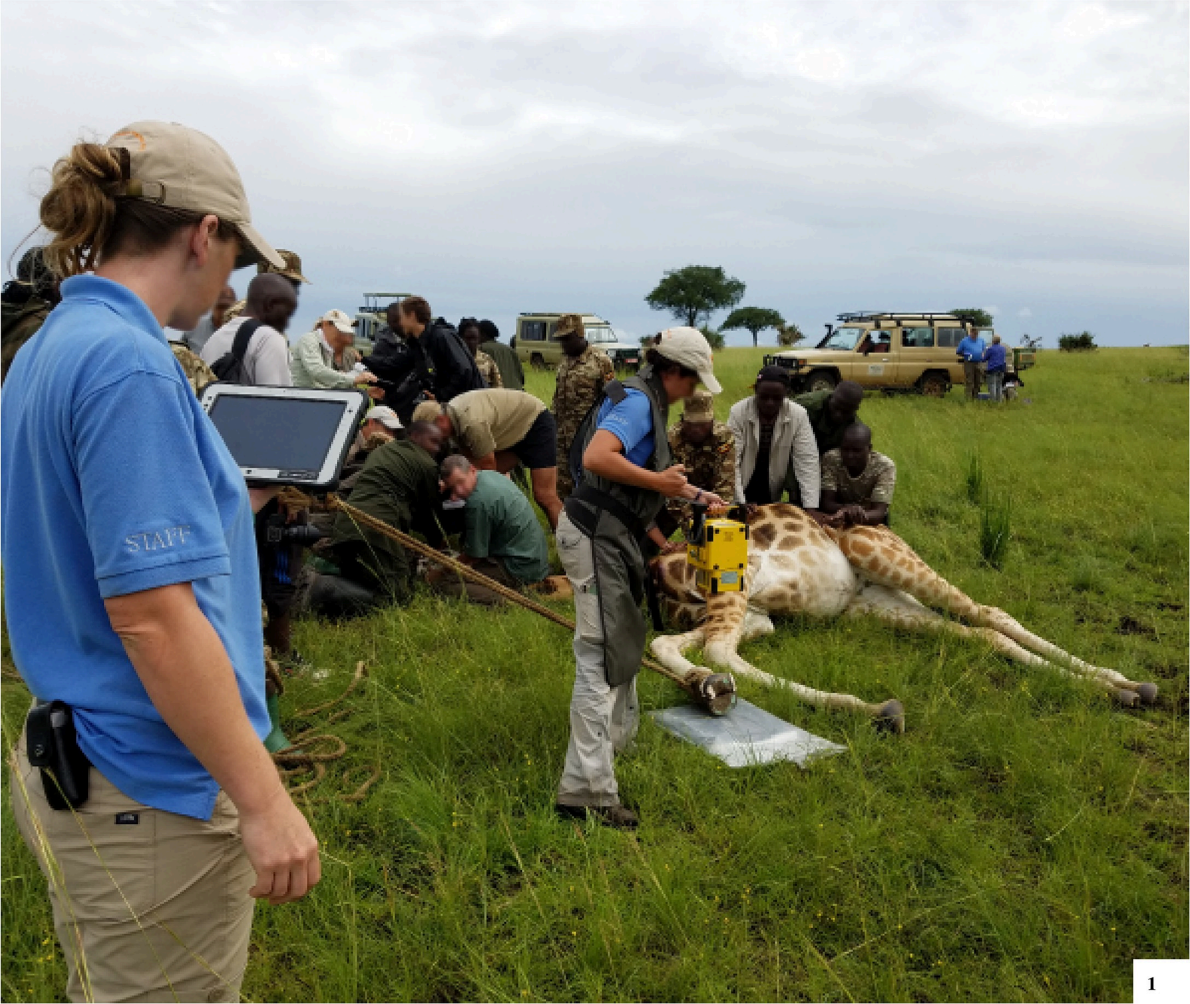
Giraffe positioning during immobilization. A leg rope was used on the giraffe for human safety during foot health evaluations and radiographs. The person holding the x-ray generator wore a leaded gown and a thyroid shield while collecting radiographic images. All other personnel were at a distance of at least two meters from the x-ray generator.

Feet were imaged with the giraffe in lateral recumbency, using either a medial or lateral oblique projection (dorsomedial-palmarolateral or dorsolateral-palmaromedial oblique; Fig 1). Radiographs were collected using portable direct-capture digital radiography (X1 ROVER tablet DR, Vet Rocket, LLC, Santa Clara, CA 95050, U.S.A.), a portable radiograph generator (MinXRay TR90B, MinXRay Inc., Northbrook, IL 60061, USA), and a wireless digital radiography panel (Canon CDXI 801C 11” X 14”, Irvine, CA 92618–3731, U.S.A.). Projections were acquired with the x-ray generator held manually using a 68- to 74-kVP and 20-mAs technique at a distance of about 40 inches (1 meter) above the foot. For safety, a leg rope was used on the giraffe and the mechanic holding the generator wore a leaded gown and thyroid shield. All other personnel were at a distance of at least two meters from the x-ray generator.

**Fig 2:**
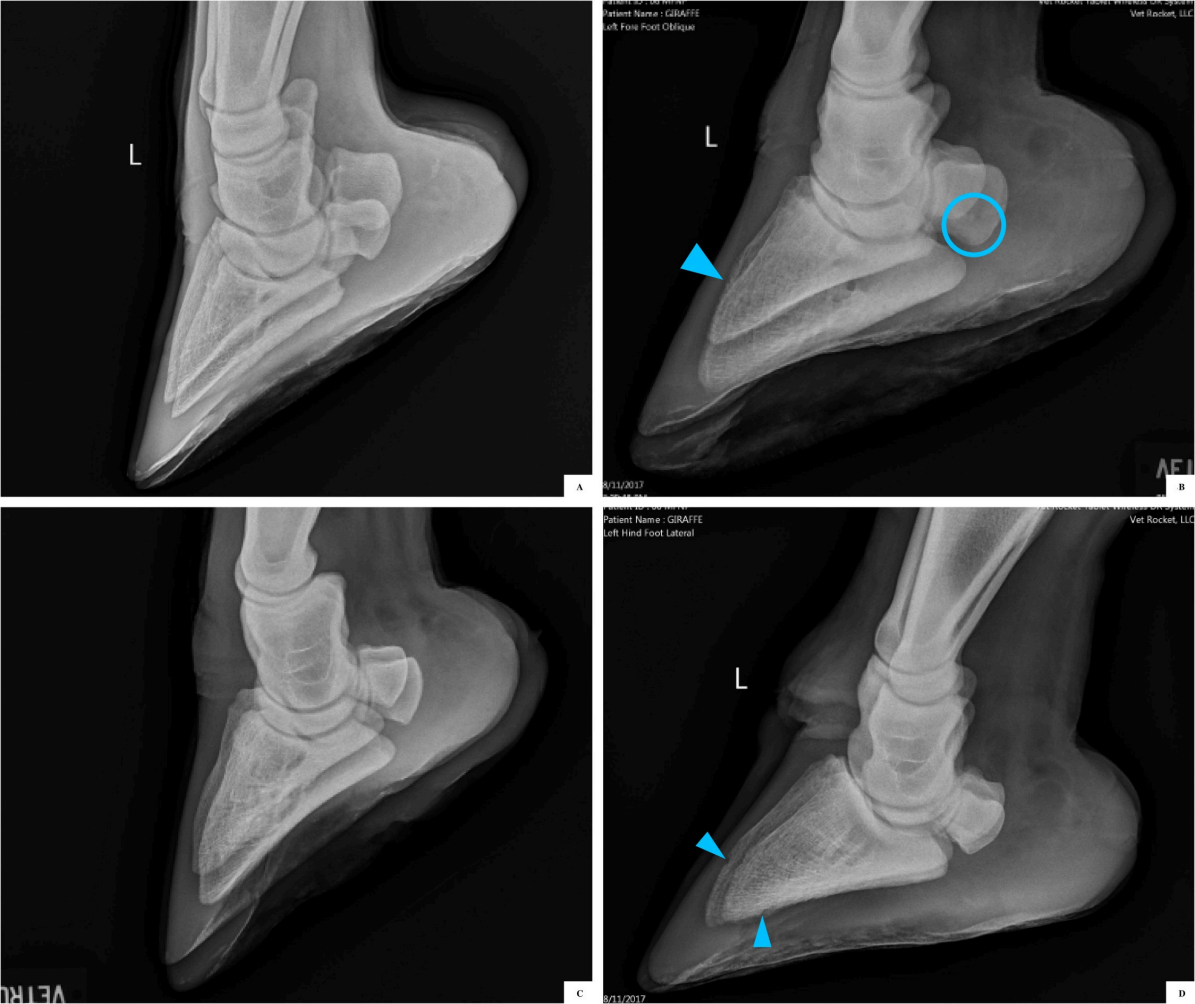
Foot radiographs from free-ranging young adult giraffe. Projections were collected with giraffe in lateral recumbency, using either a medial or lateral oblique projection (dorsomedial-palmarolateral or dorsolateral-palmaromedial oblique). (A) Normal front foot with no pedal osteitis or sesamoid cysts. (B) Abnormal front foot with mild (level 1) pedal osteitis on the dorsal surface (blue arrow) and sesamoid cysts (in circle). (C) Normal hind foot with no pedal osteitis or sesamoid cysts. (D) Abnormal hind foot with mild (level 1) pedal osteitis involving both the dorsal and plantar surfaces (blue arrow). In both the front and hind feet the sole plane runs parallel to P3 bone. Compared to the front foot, the hind foot has a more rounded toe tip of the distal phalangeal bone.

Each radiograph was evaluated by a veterinarian board-certified by the American College of Veterinary Radiology (MB) for evidence of foot pathology, and no additional information was provided about each giraffe before the images were reviewed (Fig 2). Based on pathologies and existing rating systems described for zoo giraffe [21], each digit was assessed for the presence or absence of distal interphalangeal (DIP) joint osteoarthritis (OA), distal phalangeal bone (P3) fracture, P3 osteitis, P3 rotation, and for distal sesamoid bone cysts. The severity of P3 osteitis was also rated for each digit using a scale of 0 to 3 (0 for unaffected; 1 for mild osteitis with mildly irregular solar margins; 2 for moderate osteitis with moderately irregular solar margins and/or small area(s) of bone lysis; 3 for severe osteitis with markedly irregular solar margins and/or single or multifocal medial to large area(s) of bone lysis or extensive ill-defined lysis).

**Fig 3:**
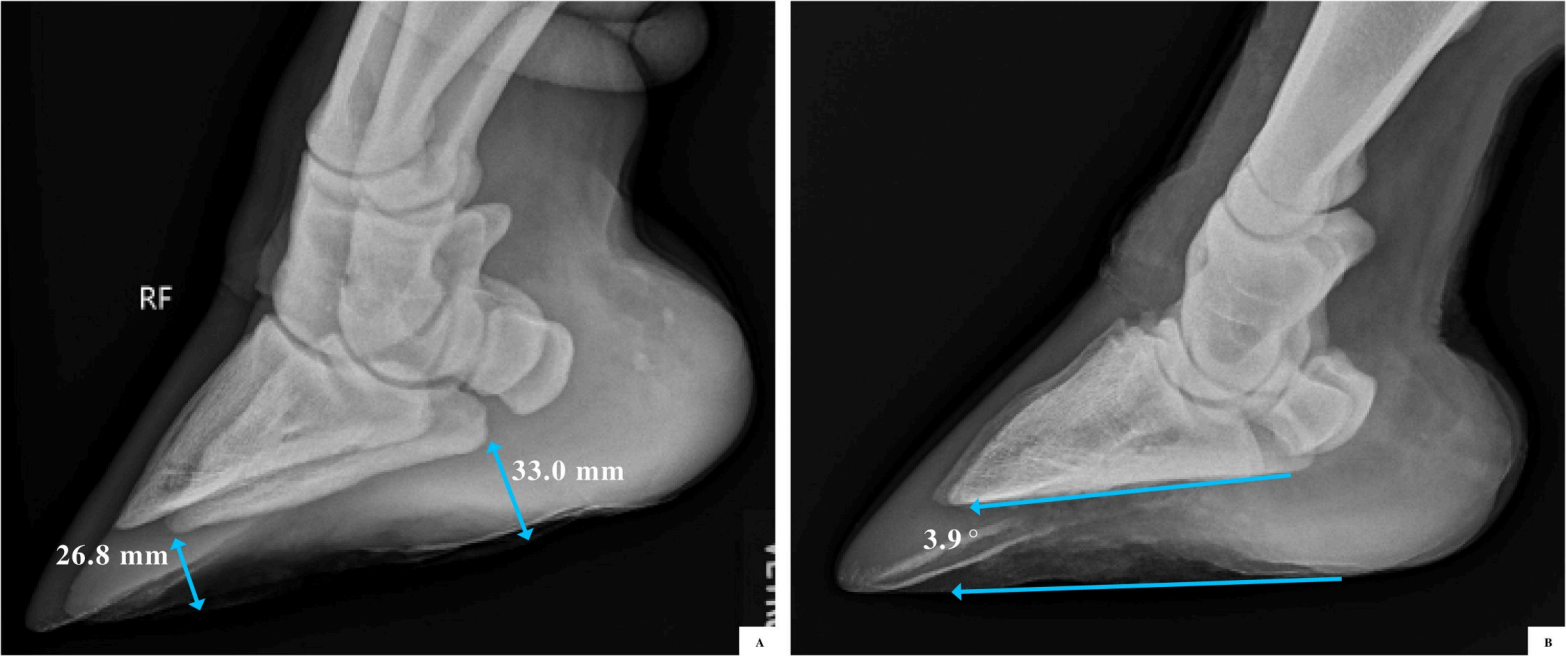
Giraffe foot sole height was thicker by the heel than by the toe tip, demonstrating a positive palmar angle. (A) Using an oblique view, sole thickness was measured from the most caudal and most distal weight-bearing aspect of P3. (B) For feet where medio-lateral or latero-medial radiographs were also available, the average palmar angle was 2.65° (range 1.88°-4.29°) and the average plantar angle was 2.26° (range 1.61°-3.23°).

Radiographs were used to assess the sole plane of the hoof, to compare the shape of P3 bones in the front vs hind feet, and to identify the palmar or plantar angle. The relative thickness of the sole was measured for each foot using an oblique view, with sole thickness measured from the most caudal and most distal weight-bearing aspect of P3 (Fig 3A). The solar aspect of P3 was rated as flat or slightly concave, and the sole plane was evaluated for how parallel it was relative to the palmar surface of the P3 bone. The palmar or plantar angle was measured from 15 front feet and 12 hind feet of 16 giraffe. This angle was calculated from medio-lateral or latero-medial radiographs by comparing the weight-bearing surface of the P3 bone with the sole surface (Fig 3B).

### Foot health assessments

**Fig 4:**
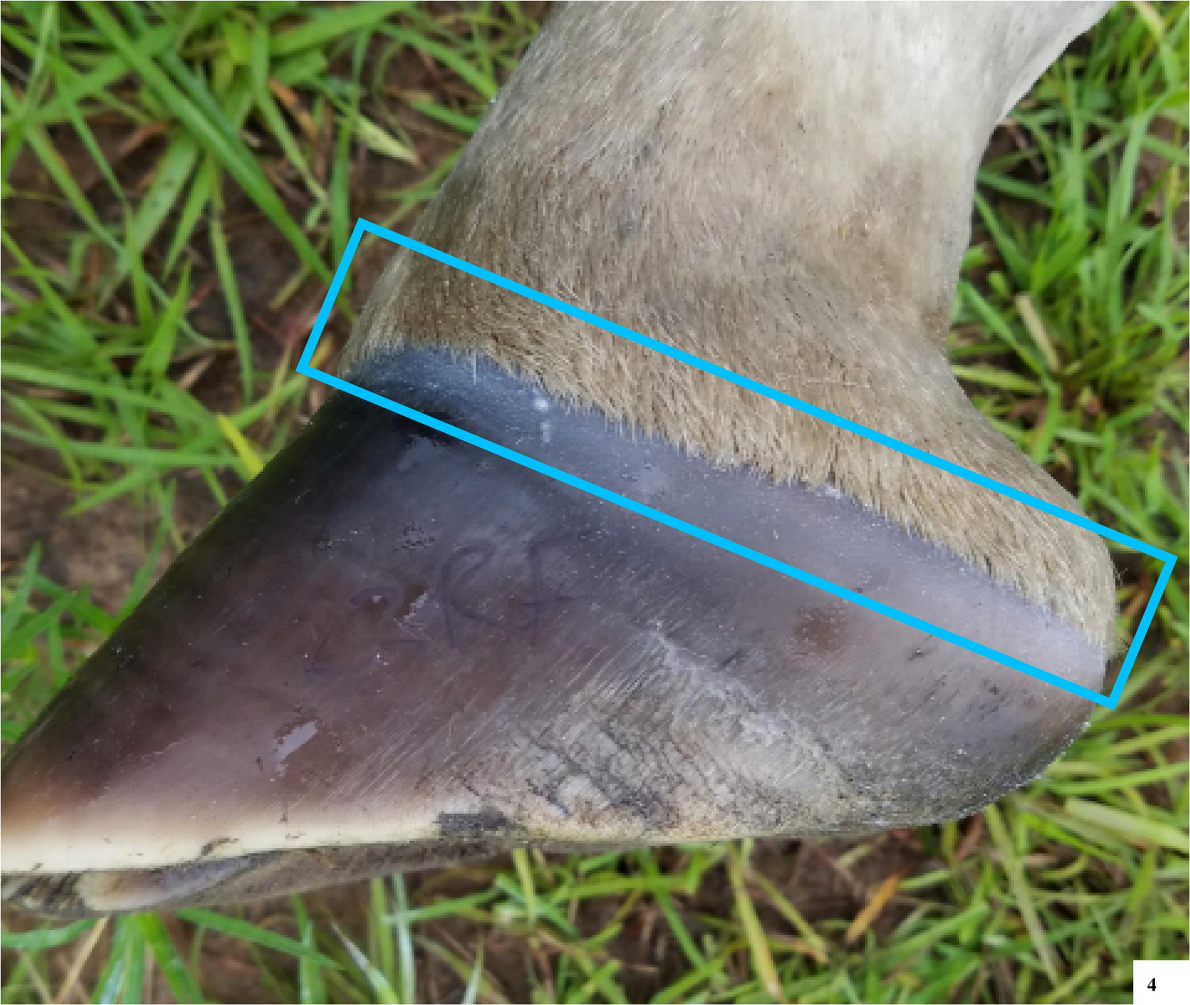
Lateral view of a giraffe front foot, showing a relatively straight coronary band.

**Fig 5:**
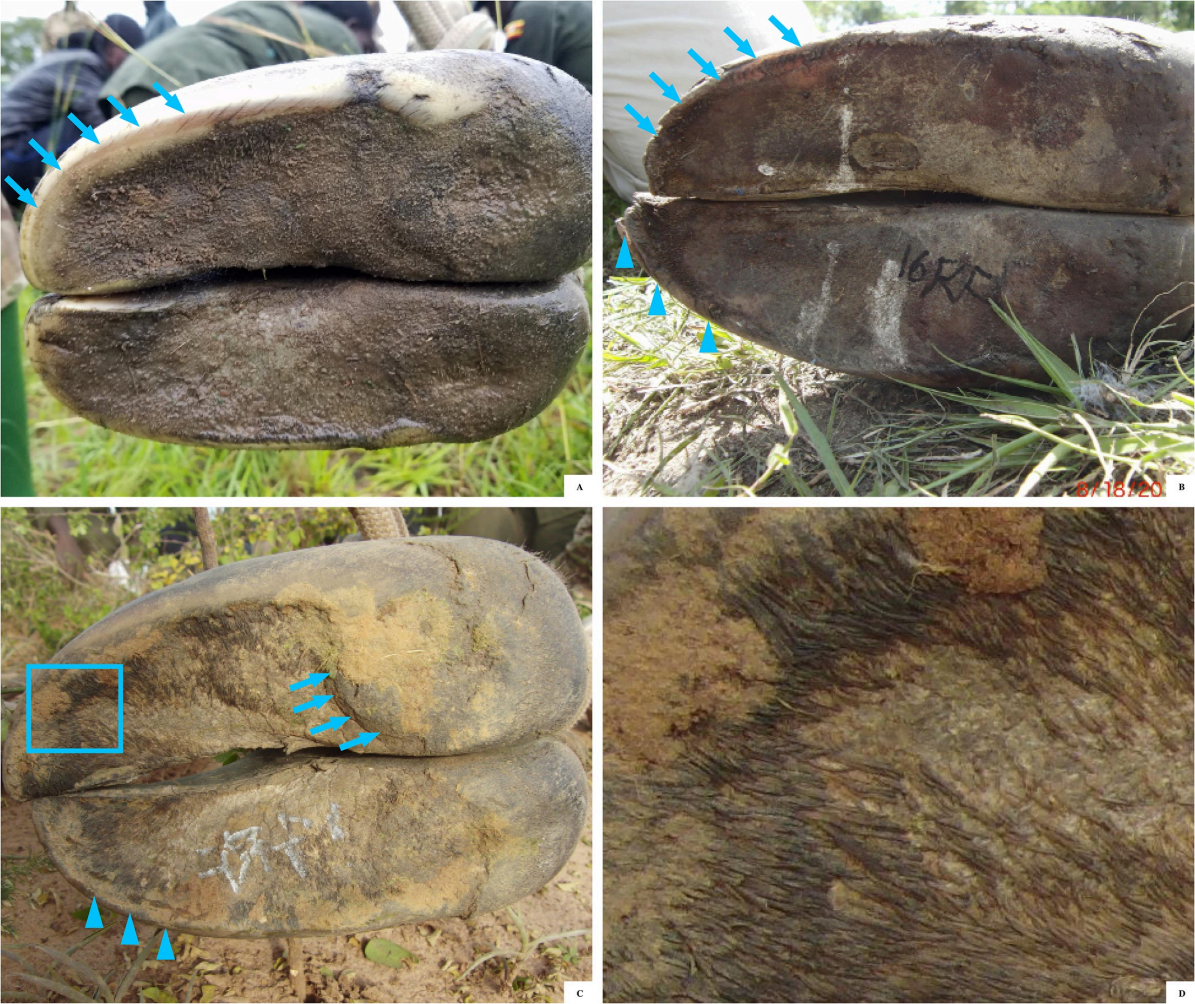
Solar views of giraffe front feet, demonstrating how hooves are self-maintained during the rainy season in Murchison Falls National Park, Uganda. (A) A well maintained balanced capsule and no hoof wall overgrowth. The wall at the toe tips and periphery (↑) is worn to the same level as the sole. (B) A foot with mild hoof wall overgrowth by the toe tip still maintains good foot symmetry medially to laterally, but has a slightly overgrown lateral toe tip (blue triangles). The medial claw hoof wall (↑) has broken or chipped off and is being worn off to the level of the sole. For the lateral claw, the hoof wall has grown beyond the level of the sole (blue triangles) and is starting to chip or break off. This suggests that many hooves are not getting much longer than moderate overgrowth before the process of exfoliation of the sole and breaking the hoof wall happens, which maintains the balance of the hoof capsule. (C) A foot with moderate overgrowth of the hoof wall, sole, and heel (↑). This foot has prominent sole papillae (□). (D) Close-up of sole papilla from 5C, which have a hair-like projections as the sole starts to overgrow.

The feet of 16 giraffe were photographed from lateral (Fig 4) and solar views (Fig 5), from two males and 14 females. Most giraffe did not have their feet photographed during the first few days of field captures as the team was prioritizing short immobilization time for patient safety while also working out logistics for collecting foot radiographs. After several days of immobilizations, foot photographs were then consistently collected on the remaining animals. Of the 56 feet that were imaged, 30 were front feet and 26 were hind feet. For lateral views, 13 giraffe had all feet assessed, two giraffe had both front feet assessed, and one giraffe had one hind foot assessed. For solar views, eight giraffe had all feet assessed (including one giraffe with partial images of one front sole), two had both front and one hind foot assessed, one had one front and both hind feet assessed, two had both front feet assessed, and one had one front foot assessed.

**Fig 6:**
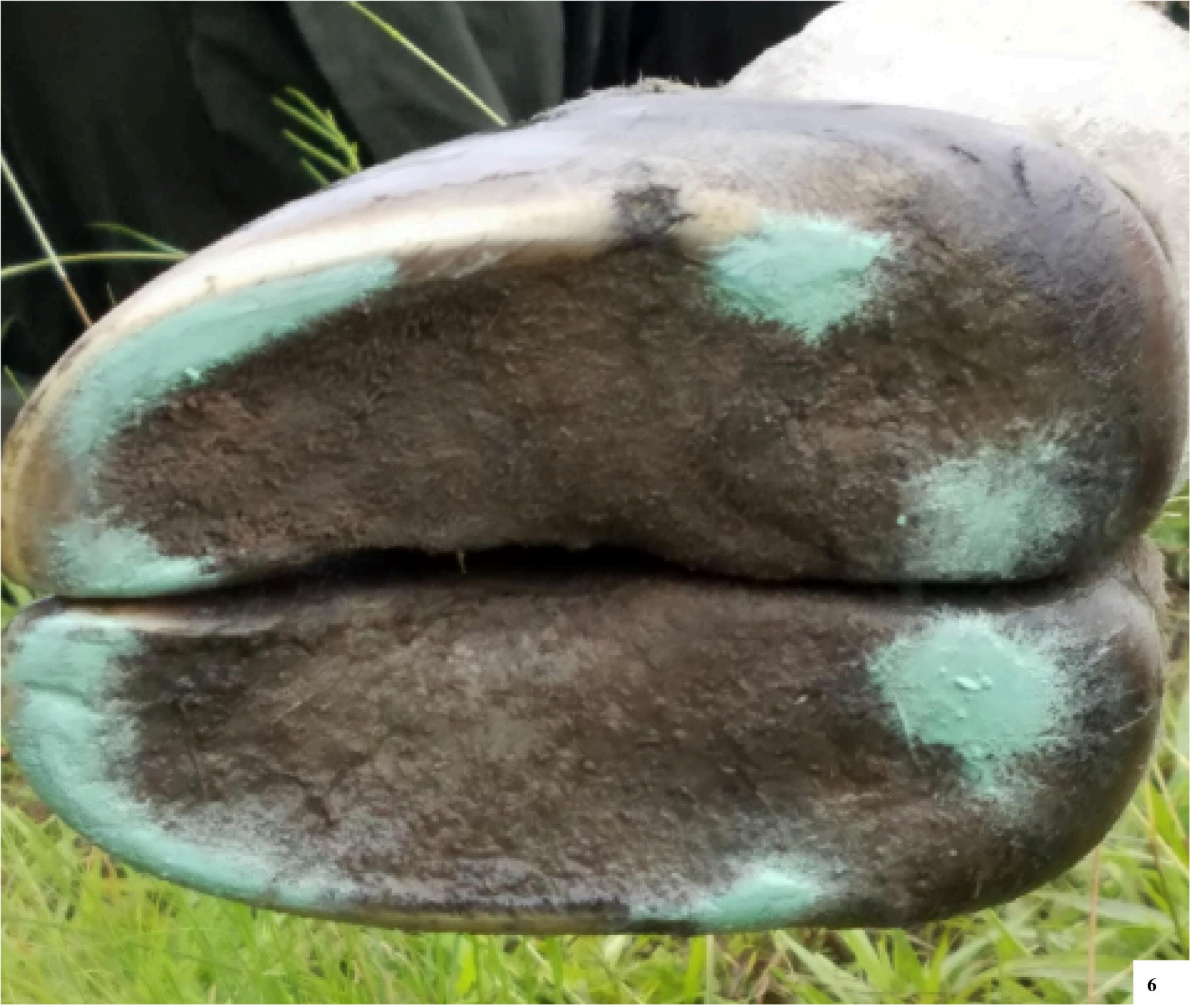
The solar surface of the giraffe’s foot is concave with hoof ground contact points around the periphery of the foot including the hoof wall, heel, and parts of the sole. Paint marks the most prominent aspects of the sole with the giraffe in lateral recumbency, with most paint on the hoof wall and heel.

The solar surfaces of the foot were evaluated and photographed for seven female giraffe (Fig 6). In this non weight-bearing position, all feet had a concave sole and tissue height by the hoof wall and heel. These hoof ground contact points of the hoof wall and heel were identified by painting a flat wooden board, rubbing the freshly painted surface along the underside of the hoof, and then evaluating which portions of the foot had been painted. Of the 20 feet evaluated, 12 were front feet and eight were hind feet. Three giraffe had weight bearing assessed for all feet, two had only front feet imaged, one had one front and two hind feet imaged, and one giraffe had only one front foot imaged.

All feet, with associated photos taken for each giraffe, were evaluated for sole abscesses, coronary band abnormalities, digit symmetry, dirt and sole foreign body impaction, and hoof overgrowth. The location and severity of overgrowth was rated for each digit using a scale of 0 to 3, with 0 for unaffected, 1 for mild, 2 for moderate, and 3 for severely overgrown digits.

## Results

### Radiographs

Radiographic evidence of foot disease was uncommon in the giraffe in this study (Table 1, S1 Table). Overall, 12.5% of the giraffe had P3 osteitis, 4.2% had sesamoid cysts, and none had DIP OA, P3 fractures, or P3 rotation.

**Table 1:** Radiographs demonstrated that foot pathologies were uncommon in these young adult free-ranging giraffe.

Foot Radiographed	Number of Feet	DIP OA	P3 Fracture	P3 Osteitis	P3 Rotation	Sesamoid Cyst
**Front**	45	0	0	3	0	2
**Hind**	41	0	0	2	0	0
**Total Feet**	86	0%	0%	5.9%*	0%	2.3%

*85 feet evaluated for P3 osteitis and 1 foot was excluded as osteitis could not be well evaluated from the available radiograph.

Pedal osteitis was identified in 12.5% (3/24) giraffe, representing both sexes (Table 2, Fig 2B). One male and one female had P3 osteitis described in one front foot each, with osteitis localized only on the palmar surface towards the toe tip. One female had osteitis in both front and one hind foot, with osteitis lesions both on the palmar and dorsal surfaces of the digits. This same female was the only giraffe with distal sesamoid cysts in her front feet (Fig 2B) but otherwise had no observed body condition and morphological difference in this individual giraffe compared with the others. For all three giraffe, when osteitis was present, it was rated as mild (level 1) [21], was present in both digits of that foot, and anatomically was not near the site of the deep digital flexor tendon attachment to P3.

**Table 2:** Foot radiographs demonstrate that P3 osteitis and distal sesamoid cysts were uncommon in both male and female free-ranging giraffe.

Sex	Number of Feet	P3 Osteitis Front Feet	P3 Osteitis Hind Feet	Sesamoid Cyst Front Feet	Sesamoid Cyst Hind Feet
**Male**	5	1	0	0	0
**Female**	19	1	2	1	0
**Total Giraffe**	24	8.3%	8.3%	4.2%	0%

In 92.9% of giraffe feet there was an observed positive palmar angle, meaning the hoof height was thicker by the heel than by the toe tip when measured from radiographs (Table 3, Fig 3). Of the 45 front feet assessed, 43 feet had a ratio <1.0 (hoof height greater at heel than toe), one foot was parallel (= 1.0), and one foot had a ratio >1.0 (hoof height greater at toe than heel). Of the 40 hind feet assessed, 36 feet had a ratio <1.0, and four had a ratio >1.0. For feet where medio-lateral or latero-medial radiograph radiographs were also available, the average palmar angle was 2.65° (range 1.88–4.29°) and the average plantar angle was 2.26° (range 1.61–3.23°).

**Table 3:** Radiographs show that the hoof was consistently thicker at the heel relative to at the toe tip in free-ranging giraffe, resulting in a positive palmar/plantar angle. Sole heights were measured from the most palmar/plantar weight bearing surface of P3 (“heel”) and from the most distal weight-bearing surface of the P3 (“toe tip”).

Foot	P3 site	Median hoof height (mm)	Min-max hoof height (mm)	Ratio hoof height heel to toe	Min-max ratio height heel to toe
**Front**	Heel	34.00	21.60–43.40	0.84	0.61–1.01
	Toe tip	28.80	19.50–39.20		
**Hind**	Heel	30.00	24.80–49.70	0.86	0.58–1.04
	Toe tip	25.80	14.40–37.60		

Radiographs demonstrated that the sole plane was parallel to the P3 bone. The solar aspect of P3 was rated as flat in 87.2% of feet (75/86 feet) and had mild concavity in 12.8% of feet (11/86 feet). When comparing front and hind feet, P3 was slightly more rounded at the toe tip in hind feet when compared with front feet.

### Foot health assessments

Advanced foot disease was not present in the giraffe in this study (Table 4). No giraffe had coronary band abnormalities or sole abscesses and only one giraffe had an old injury to a hind foot. In that individual, the medial claw hoof wall had an old scar.

**Table 4:** Photographs demonstrate that hoof and heel overgrowth and dirt impaction of the sole was regularly present but foot pathologies were uncommon in free-ranging giraffe.

Foot Photographed	Hoof Wall Overgrowth	Heel Overgrowth	Papilla Growth in Sole	Dirt Impaction in Sole	Coronary Band Abnormalities	Sole Abscess	Hoof Wall Injury
**Front**	6 of 25	4 of 25	5 of 25	8 of 26	0 of 30	0 of 25	0 of 25
**Hind**	5 of 21	6 of 21	2 of 21	4 of 21	0 of 26	0 of 21	1 of 21
**Total Feet**	23.9%	21.7%	15.2%	25.5%%	0%	0%	2.2%

Overall, 43.5% (20/46) of feet had hoof overgrowth and that overgrowth was rated as mild in almost all cases. Of the 46 feet evaluated from the solar aspect, 23.9% (11 feet) had hoof wall overgrowth by the toe, 21.7% (10 feet) had heel overgrowth, and 15.2% (seven feet) had papillary growth from the sole (Table 4). Similar numbers of front and hind feet were overgrown in each of the different parts of the foot evaluated (Table 5), and hoof overgrowth was predominantly rated as mild to moderate. When sole overgrowth was present, it was seen as small amounts of papillary growth in the rostral half of the foot. One female had marked overgrowth of hoof walls, moderate overgrowth of heels, and mild papillary growths present in the soles of all four feet.

**Table 5:** Photographs of the solar surfaces demonstrated hoof overgrowth in both the front and hind feet of free-ranging giraffe. When sole overgrowth was observed, the underside of the foot remained concave but sometimes had small amounts of papillary growth.

Foot photographed		Hoof wall overgrowth	Heel overgrowth	Sole with papillary growth
**Front**	Medial digit	0/25	0/25	0/25
	Lateral digit	2/25	1/25	2/25
	Both digits	4/25	3/25	3/25
**Hind**	Medial digit	0/21	1/21	0/21
	Lateral digit	0/21	3/21	1/21
	Both digits	5/21	2/21	1/21
**Total feet**		**11/46**	**10/46**	**7/46**

The ground contact points of the hoof wall and heel were along the periphery, including hoof wall, parts of the heel, and edge of the sole (Fig 5). The sole was consistently concave and paint did not adhere to this central portion of the foot.

Foot symmetry was subjectively evaluated and suggested overall good symmetry in toe length, sole width, and hoof height between medial and lateral digits for each foot. While the hoof wall was relatively short, it was slightly longer by the toe tip. Compared to front feet, hind feet were narrower, straighter, and less rounded at the toe. Two giraffe had marked asymmetry between medial and lateral digits of one hind foot that appeared associated with abnormal conformation and not hoof overgrowth. One of these giraffe had mild heel overgrowth on the opposite hind foot, the other giraffe had no hoof overgrowth on the other feet.

Overall, 37.5% (6/16) giraffe had mild dirt compaction in the soles of their feet. Of these, only two giraffe had mild hoof overgrowth, the remaining had no hoof overgrowth.

## Discussion

This study is the first to describe foot shape and radiographic findings in the feet of free-ranging young-adult giraffe and identified a low prevalence of foot disease. Although this single population of giraffe might not reflect the conditions of other free-ranging giraffe, it offers insight into possible factors associated with lameness and foot disease in zoo giraffe and other exotic hoofstock species.

### Radiographs

Radiographic evidence of foot disease was uncommon in this study. While studies of feral horses indicate that wild foot studies are not always an ideal model for how the foot should look when self-maintained with substrate, diet, and exercise [35], the observation that foot disease was uncommon in these giraffe suggests the current study could be a useful reference.

Common foot pathologies in zoo giraffe were uncommon or not present in the free ranging giraffe. We observed that in young adult giraffe, 12.5% had pedal osteitis, 4% (1 animal) had sesamoid cysts, and none had DIP OA, P3 rotation, or P3 fractures. In comparison, in one study of zoo giraffe, 86% of giraffe had front foot pedal osteitis, 72% had osteoarthritis of the distal interphalangeal joint, 59% had sesamoid cysts, 53% had P3 rotation with a negative palmar angle, and 36% had P3 fractures of the front feet [21]. Additionally, all zoo giraffe in that study had osteoarthritis in at least one digit by seven years old and developed P3 fractures as young as 10 years old [21]. While these two studies were not well age-matched, the presence of pedal osteitis and a negative palmar angle in relatively young zoo giraffe suggest early onset differences in foot health. Additionally, while differences in foot health in horses and cattle have been described based on subspecies, diet, exercise, and flooring [36–42], it is currently undetermined how important these factors may be for giraffe.

The giraffe in this study all had positive palmar and plantar angles from 1–5°, with the sole consistently thicker by the heel of the foot compared with the toe tip. While this was measured in a non weight-bearing position, the angles are similar to what has been described for the normal weight-bearing equine foot with an angle of 2–5° [43]. In horses, the heel to toe height ratio and the angles of the distal phalanx are also well correlated with how the foot is mechanically loaded [44] and are useful for evaluating laminitis [45]. Reference ranges for palmar and plantar angles or hoof height ratios in giraffe have not previously been available but could be useful in identifying factors of foot disease in zoos. Ideally, radiographs would be obtained when the foot is weight-bearing, as relative hardness of the sole could influence the weight-bearing heights; however, this type of study is impractical in these free-ranging giraffe.

Abnormalities in palmar/plantar angles are associated with lameness and foot pathologies in domestic hoofstock and sometimes in zoo giraffe. In domestic horses, a low or negative palmar or plantar angle has been associated with foot pathologies, particularly of the deep digital flexor tendon and navicular apparatus, and lameness [46] while an excessive positive palmar angle can be associated with laminitis [47]. In horses, Negative Palmar Angle Syndrome (NPAS) is associated with progressive heel overgrowth and collapse [48]. NPAS in horses can be graded to help define how the hoof is trimmed and possibly the shape of shoe needed to restore more normal weight bearing and thus address lameness [48]. While at least one zoo giraffe has been described with an excessive positive palmar angle with parturition-associated laminitis [9], some zoo giraffe lameness cases appear to be associated with a negative palmar angle [21] that could resemble NPAS in horses. Further study is warranted to determine if hoof trimming that maintains a slightly positive palmar/plantar angle as observed in free-ranging giraffe could help zoo giraffe and other exotic hoofstock species better maintain foot health and avoid some lameness.

In this study, the weight-bearing surface of P3 was parallel to the sole of the hoof. This suggests that once the sole plane of the hoof has been exfoliated and identified, it could be a useful landmark for trimming the hoof without radiographs.

### Foot shape and health

Giraffe feet hoof ground contact points were along the hoof wall, parts of the heel and the edge of the sole, with the sole having a concave shape. As the foot is a dynamic structure, additional parts of the hoof may contact the ground when the giraffe begins to load bear on that foot. Hoof ground contact points along the periphery of the foot are similar to the horse foot maintaining slight height of the hoof walls, with or without a shoe, and a dynamic sole structure designed to adapt to variable terrain and seasonal changes [49]. Notably, feral horses from both soft or hard substrate habitats had greater mean sole depth than domestic Thoroughbred horses [50]. This suggests differences in sole thickness or foot shape associated with zoo substrate, diet, exercise, or other factors could also affect giraffe foot function and gait.

The observation that free-ranging giraffe have a concave sole, no overgrowth in the central areas of the sole, and no radiographic pathologies in that portion of P3 demonstrates how important the differences in foot shape may be between zoo and free ranging giraffe. In horses, as assumed in giraffe, while the periphery of the foot is taller, the sole of the foot also has a supporting role in weight bearing with pressure, release, and shock absorption with each step [49]. With sole overgrowths, the foot loses this concave shape and will consistently bear weight and have pressure along the sole of the foot, as is described in some zoo giraffe [21]. Foot pressure studies from domestic bovine claws identified the highest pressure when standing is actually on the sole of the foot and not on the hoof wall [51]. These same parts of the sole/heel region that carry the maximum pressure in domestic bovines are also parts of the foot relatively susceptible to injuries [51]. Similarly, zoo giraffe with sole overgrowth have an association between sole overgrowth near the heel of the foot and the location of pedal osteitis and P3 fractures [21] which suggests a similar process of injury as the foot overgrows and loses its concave shape.

The study describes normal growth and wear patterns for free-ranging giraffe hooves that are self-maintaining in a natural habitat during the intermediate rainy season with relatively soft substrate in the grasslands and woodlands of MFNP. Subjectively, these feet had overall good symmetry. However, the hoof wall was slightly taller than the sole plane, especially in the toe tip. A similar pattern of longer toe tip length has been described in feral horses that self-maintain their feet in a softer environment, while more toe tip wear has been described when feral horses are primarily on harder surfaces [50]. This can serve as a point of comparison when evaluating zoo giraffe feet kept on surfaces such as concrete which are known to be associated with foot problems in other megaherbivores such as elephants [52, 53] and rhinos [54].

The coronary band was relatively straight when viewed from the lateral surface, similar to what has been described for healthy horse feet. The shape of the coronary band is significantly different between limping and non-limping horses [55]. While no data on coronary band shape is available for zoo hoofstock species, this could be a useful non-invasive technique to help evaluate foot health in zoos.

## Conclusions

1. This study is the first to describe the concave foot shape, positive palmar/plantar angle, and radiographic anatomy of free-ranging giraffe, and will serve as a reference for further study.
2. The free-ranging giraffe in this study had a low prevalence of foot pathologies. Based on radiographs, pedal osteitis and distal sesamoid bone cysts were uncommon and no giraffe had distal interphalangeal joint osteoarthritis, distal phalangeal bone rotation, or distal phalangeal bone fractures.
3. This study suggests qualitative differences in foot shape, foot health, radiographic anatomy, and foot pathologies when comparing free-ranging and zoo giraffe. These differences may be significant factors in the prevalence of foot diseases in zoo giraffe and suggests there are likely benefits for zoo giraffe to have routine foot assessments and hoof trims.

## Supporting information

S1 Table The feet of 24 Nubian giraffe were evaluated for radiographic evidence of disease. In these giraffe, pedal osteitis and sesamoid bone cysts were relatively uncommon (3/24 giraffe with osteitis, 1/24 giraffe with sesamoid cysts), and no giraffe in the study had osteoarthritis of the distal interphalangeal joint, distal phalangeal bone (P3) fractures, or P3 rotation.
(XLSX)
